# Relation between mannose-binding lectin (MBL) gene codon 54 polymorphism (allele B) and susceptibility to HTLV-1 infection

**DOI:** 10.1186/1742-4690-11-S1-P80

**Published:** 2014-01-07

**Authors:** María Saliba Pineda, Mirta Remesar, María Belén Bouzas, Ariel Fridman, Carlos Remondegui, Paula Aulicino, Luisa Sen, Andrea Mangano

**Affiliations:** 1 Laboratorio de Biología Celular y Retrovirus- CONICET, Hospital Garrahan, Ciudad Autónoma de Buenos Aires, Argentina; 2 Servicio de Hemoterapia, Hospital Garrahan, Ciudad Autónoma de Buenos Aires, Argentina; 3 Unidad de Virología, Hospital Muñiz, Ciudad Autónoma de Buenos Aires, Argentina; 4 Laboratorio Central de Jujuy, Jujuy, Argentina; 5 Servicio de Infectología y Enfermedades Tropicales, Hospital San Roque, Jujuy, Argentina

## 

Mannose-binding lectin (MBL) plays an important role in the innate immune defense against invading microorganisms. Deficiency of functional MBL is linked to polymorphisms in the *MBL2* gene. The aim of the study was to determine the influence of MBL2 polymorphisms in susceptibility to HTLV-1 infection. A total of 43 HTLV-1 infected subjects and 127 healthy controls were evaluated for polymorphisms in the coding region of MBL2 gene. The point mutations in exon 1 at codon 54 (allele B) and codon 52 (allele D) and the wild type allele A were detected by PCR-RFLP. The frequency of the allele A, B and D was 66%, 29% and 5% among HTLV-1 infected subjects and 79%, 18% and 3% among healthy controls, respectively. Genotype and allele frequencies were statistically different between both groups, being the allele B more frequent among HTLV-1 infected subjects than in controls (29% and 18%, respectively; p=0.032). Moreover, the homozygous genotype BB was observed in 14% of HTLV-1 patients and only 3% of controls (p=0.016), and it was associated with an almost five-fold higher risk of HTLV-1 infection (p=0.016; OR=4.98, 95%CI=1,33-18,63). Our results suggest that carriers of the MBL2 allele B are more susceptible to HTLV-1 infection. Further studies with a large number of individuals are ongoing to confirm the impact of MBL polymorphisms as genetic determinant of HTLV-1 susceptibility.

